# A fully integrated duplex RT-LAMP device for the detection of viral infections

**DOI:** 10.1007/s10544-023-00676-w

**Published:** 2023-09-08

**Authors:** Nicolas Mytzka, Skaiste Arbaciauskaite, Natalia Sandetskaya, Kai Mattern, Dirk Kuhlmeier

**Affiliations:** 1 MicroDiagnostics Unit, Fraunhofer Institute for Cell Therapy and Immunology, 04103 Leipzig, Germany; 2 Institute of Cell Biology and Neurobiology, Charité—Universitätsmedizin Berlin, Charitéplatz 1, 10117 Berlin, Germany

**Keywords:** SARS-CoV-2, Influenza, Coronavirus, Respiratory infections, Point-of-care, RT-LAMP, Multiplex

## Abstract

Respiratory viruses can cause epidemics or pandemics, which are worldwide outbreaks of disease. The severity of these events varies depending on the virus, its characteristics, along with environmental factors. The frequency of epidemics and pandemics caused by respiratory viruses is difficult to predict, but the potential severity of such events underlines the importance of continued monitoring, research, and preparation for emerging infectious diseases. To help improve pandemic preparedness, we created a fully integrated duplex reverse transcription loop-mediated isothermal amplification (RT-LAMP) device targeting two respiratory viruses, influenza A/X-31 virus and bovine coronavirus, as a replacement for SARS-CoV-2. This device can be adapted to any other respiratory virus. In this study, we showed and evaluated a prototype of a microfluidic system, and showed that duplex RT-LAMP can detect and distinguish between the two viruses, with LoDs of 2,000 copies/ml for bovine coronavirus and 200 copies/ml for influenza A/X-31 virus.

**Graphical abstract:**

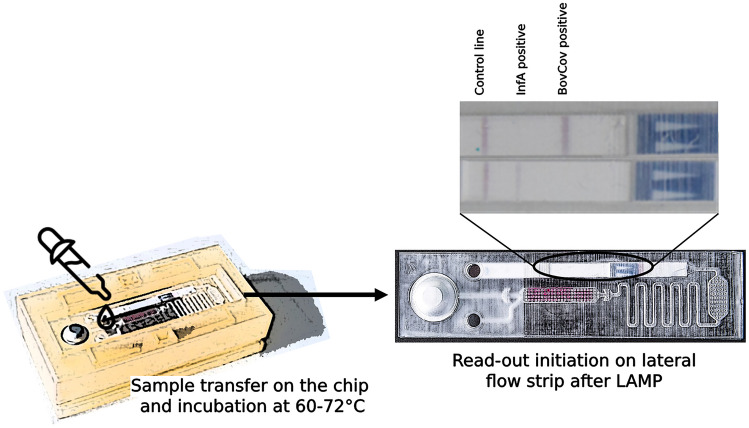

## Introduction

Respiratory viruses have the potential to cause epidemics or pandemics, which are widespread outbreaks of disease (Abdelrahman et al. 2020). The frequency of these events can vary depending on the specific virus that is responsible for the disease and its characteristics, as well as environmental factors (Azziz Baumgartner et al. 2012; Leung 2021). Respiratory viruses, such as the seasonal influenza virus, cause annual epidemics that result in millions of cases and hundreds of thousands of deaths worldwide (Weinstein et al. 2003; Iuliano et al. 2018; Javanian et al. 2021). Moreover, some respiratory viruses can also cause pandemics - global outbreaks of disease that can affect a significant proportion of the world population e.g., the most recent COVID-19 pandemic caused by the SARS-CoV-2 virus (Hu et al. 2021). The frequency of epidemics and pandemics caused by respiratory viruses is difficult to predict, but the potential severity of such events underlines the importance of continued monitoring, research, and preparation for emerging infectious diseases (Biggerstaff et al. 2014; McCloskey et al. 2014; Vieira et al. 2022).

Most respiratory viruses present a very similar clinical picture, hence it is important to differentiate between prevalent viruses for accurate diagnosis and treatment (Nam and Ison 2019; Wiersinga et al. 2020; Zhang et al. 2021; Uyeki et al. 2022). Therefore, we designed an integrated molecular duplex respiratory virus test that can simultaneously detect and distinguish two viruses in a single test. Testing for two viruses in one reaction increases the scope and efficiency of diagnostics. Indeed, it helps ensure that a correct diagnosis is made, since symptoms for certain infections can be, as mentioned, similar. Preventing misdiagnoses, in turn, can reduce the provision of inappropriate treatments that could delay recovery. Secondly, performing duplex tests reduces the overall number of tests that would need to be performed, helping save time and resources, thus making duplex testing potentially more resource- and cost-effective than single pathogen targeted tests (Jiang et al. 2020; Du et al. 2021; Brachmann et al. 2023). When trying to monitor multiple infections e.g., in the case of combined testing for COVID-19 and influenza, duplex testing may be especially helpful for public health officials in tracking the spread of different illnesses. The tracking of multiple infections can be particularly important during flu season, when it can be critical to know if an outbreak is caused, and to what extent, by COVID-19 or by the flu. Indeed, the WHO has explicitly highlighted the risk of co-circulating COVID-19 and seasonal influenza for vulnerable populations (*Joint statement: Working together towards COVID-19 and seasonal influenza vaccinations for this winter* 2022). Richer epidemiological data facilitated, e.g. by more efficient duplex testing, also can help with preparedness for future epidemic outbreaks. Better data allows for the improved identification of patterns in how different illnesses spread and can thereby even help in investigations on how different infectious illnesses can best be respectively controlled.

Polymerase chain reaction (PCR) remains the gold standard for respiratory virus detection, and there are examples of multiplex panels detecting different respiratory viruses by RT-PCR (Brittain-Long et al. 2008; Anderson et al. 2013; Ni et al. 2021; Shu et al. 2022). However, performing single or multiplex reverse transcription polymerase chain reaction (RT-PCR) is generally expensive, time consuming, and personnel intense. An alternative to RT-PCR is reverse transcription loop-mediated isothermal amplification (RT-LAMP or just LAMP). A LAMP reaction can be completed in about 30 min and requires no temperature cycling (Notomi et al. 2000). Furthermore, LAMP allows for various read-out options, such as fluorescence, electrochemical, or probe-based methods combined with real-time or endpoint detection (Wong et al. 2018; Becherer et al. 2020; Moehling et al. 2021). Moreover, LAMP does not require special lab infrastructure and can be integrated in point-of-care (PoC) solutions. Finally, clinical studies demonstrate the high accuracy and efficiency of LAMP when used for respiratory virus detection (Ahn et al. 2019; Arbaciauskaite et al. 2022; Lu et al. 2022; Pu et al. 2022). However, there are very few examples in the literature of duplex or multiplex LAMP PoC tests. One of them was developed by Lee et al. - an RT-LAMP-based multiplex assay aiming to detect SARS-CoV-2, influenza A, and B in a PoC setting. The assay detected viral RNA reliably with a LoD of 50 copies/μL. Saliva samples analyzed through quantitative RT-PCR confirmed COVID-19 diagnosis in the patients that were used to validate the assay clinically. The multiplex panel was in 100% concordance with PCR testing (Lee et al. 2021). The main drawback of such a set-up is the colorimetric read-out, which is hard to interpret for some users, therefore we created a set-up with a lateral flow strip for read-out.

The main objectives of this study and our experimental set-up were: 1) to demonstrate the feasibility of duplex LAMP on a lateral flow test (LFT) 2) to integrate all the steps needed for LAMP diagnostics on a chip and to make the test easy to perform without laboratory infrastructure 3) to evaluate the performance of an integrated duplex LAMP on a chip.

## Results

### Microfluidic RT-LAMP chip design

Essential features of PoC tests are the rapid detection of their specific targets and their device-independency. Here, we designed an instrument-free, stand-alone prototype for the detection of two respiratory viruses – influenza A/X-31 virus (InfA) and bovine coronavirus (BovCov) as a BSL-2 laboratory-feasible replacement for SARS-CoV-2.

**Fig.1:**
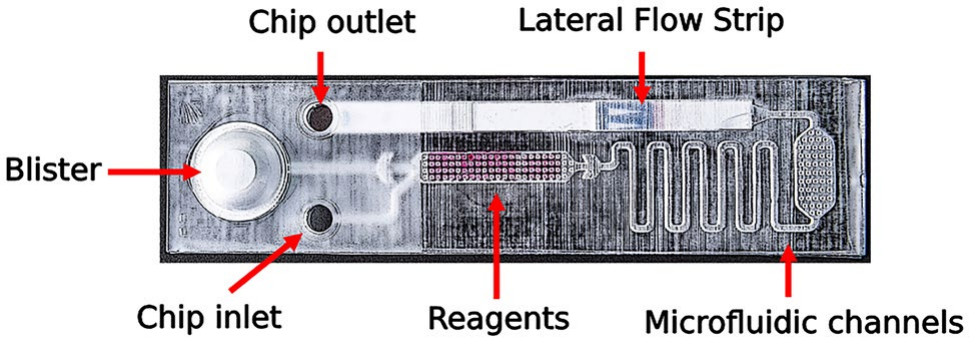
Microfluidic chip design of the duplex RT-LAMP device. Chip design with microfluidic channels, connecting the reaction chamber that contains lyophilized LAMP reagents (colored with phenol red) with the inlet, the integrated LFT and the water-filled blister

The microfluidic structures on the chip allow sample loading, RT-LAMP reaction, and subsequent LFT detection of the amplification product. The chips were produced in several iterations by 3D-printing of the embossing forms, and subsequent hot embossing of the chip molds (Fig. 1).

There are three main parts integrated in the microfluidic chip design. First, a 50 µl reaction chamber containing the lyophilized reagents for RT-LAMP (colored with phenol red for better observation of fluidic processes) and an inlet for sample loading. Second is a cavity with an outlet containing an LFT for the detection of amplified LAMP products. Third, a blister filled with water for the lateral flow assay. All parts are connected to the reaction chamber by a microfluidic channel system.

The workflow can be summarized as follows: an aqueous sample is added into the inlet of the chip, flowing to the reaction chamber by capillary forces, thereby resolving the lyophilized LAMP reagents. The RT-LAMP reaction can then be performed at a temperature between 60–72 °C (for *Bst* 2.0 polymerase) e.g., on a heating plate or similar heating system. Subsequently, the blister is pressed flushing the reaction mixture through the microfluidic channel to the LFT, initiating lateral flow detection. After 20–30 min, the LFT is ready for read-out.

### Duplex RT-LAMP

The first challenge of this detection system was the development of two compatible LAMP reactions for InfA and BovCov. Two newly designed primer sets were combined in one RT-LAMP reaction mixture for the simultaneous amplification and detection of both LAMP products. Therefore, one primer of the respective primer sets was labeled with biotin for BovCov and with digoxigenin for InfA. These two labels bind to different areas on the LFT, allowing differentiation of both LAMP products.

After sample loading, the lyophilized reagents resolved in less than one minute, allowing fast processing of multiple samples. After incubation at 60–72 °C for 30 min, the LFT was initiated by pressing the blister. In about one out of ten chips, the liquid cross-flowed in unintended directions during this process, bypassing the microfluidic channel system. This was caused by irregularities of the chip surface due to the utilized production method. These chips with incorrect microfluidic flow were excluded from evaluation and the respective experiments were repeated.

**Fig. 2:**
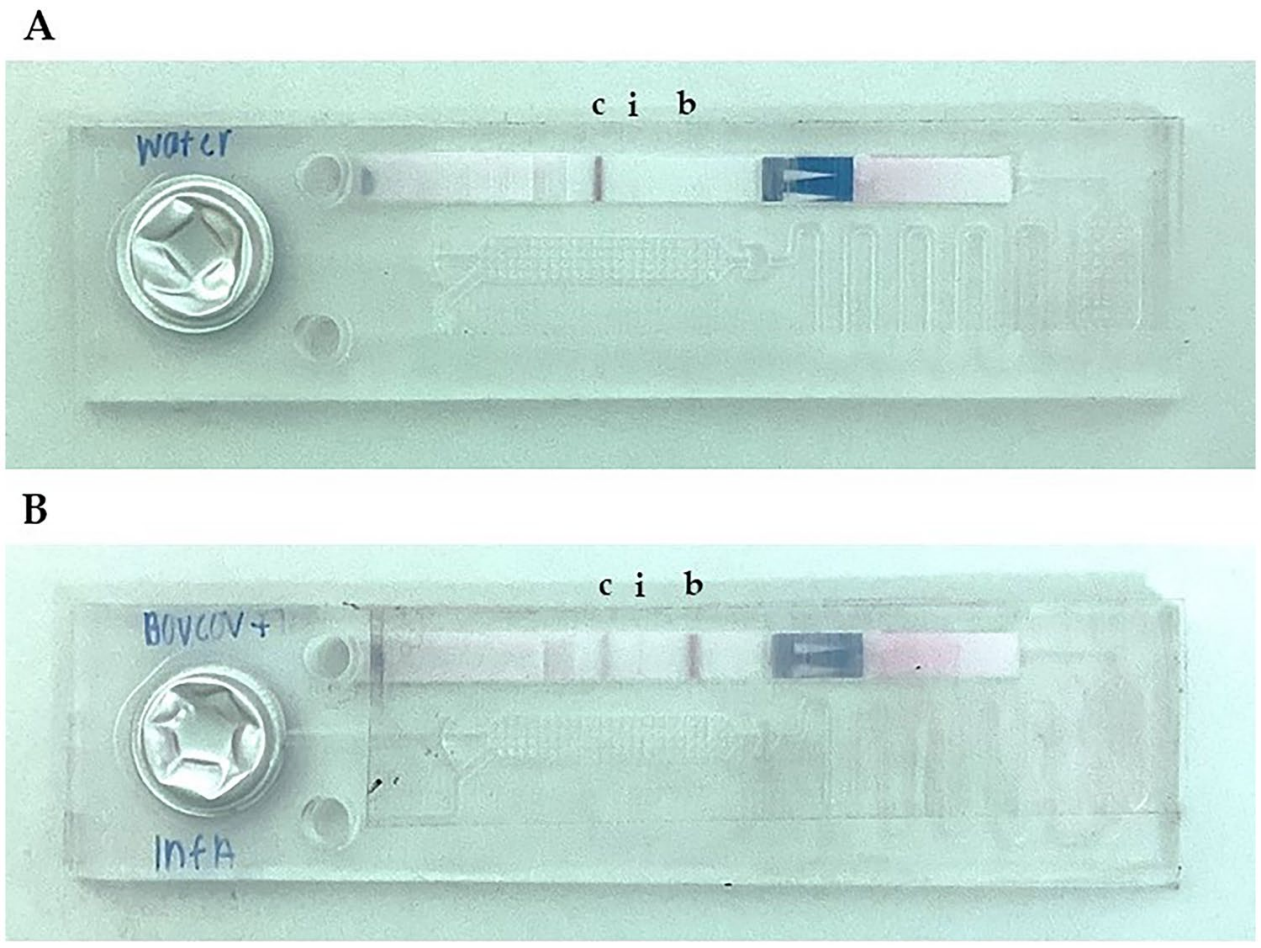
Duplex RT-LAMP assay for influenza A virus and bovine coronavirus. LFT bands from left to right: control line (c), InfA test line (i), BovCov test line (b); **A** Negative control with water; **B** Positive control with 1,000 RNA copies of InfA and BovCov

Without target RNA, only the control band was visible on the LFT confirming the absence of any specific LAMP products (Fig. 2A). In the presence of 1,000 RNA copies of both targets, three bands were observed: an intense test band for the BovCov (Fig. 2B, b), a less intense control band (Fig. 2B, c) and a weak test band for InfA (Fig. 2B, i). Hence, the amplification products of both primer sets were successfully detected after the duplex RT-LAMP reaction.

The less intense InfA band could be caused by a more efficient BovCov LAMP reaction compared to InfA LAMP reaction since both compete for the same reagents. This difference in efficiency could be reduced by adjusting the concentration of both primer sets in further development. Also, the area of the biotin-binding BovCov band is larger than the digoxygenin-binding InfA band, making the BovCov band easier to recognize.

Additionally, the slightly opaque material of the chip hindered the clear view of the LFT. This problem can be eliminated by changing the production method or material of the microfluidic chip e.g., production by injection molding.

### Limit of detection for RT-LAMP

A high sensitivity is a crucial feature of diagnostic tests for viral infections. The limit of detection (LoD) for both RT-LAMP reactions was therefore determined using purified RNA with known copy numbers. tenfold dilution series of RNA were tested with the duplex RT-LAMP device. The RNA dilutions were tested separately due to the competing behavior of the BovCov LAMP reaction mentioned previously. Additionally tenfold dilution series were tested with quantitative RT-LAMP using the LightCycler 480 system (Roche, Grenzach-Wyhlen, Germany) as a reference.

**Fig. 3:**
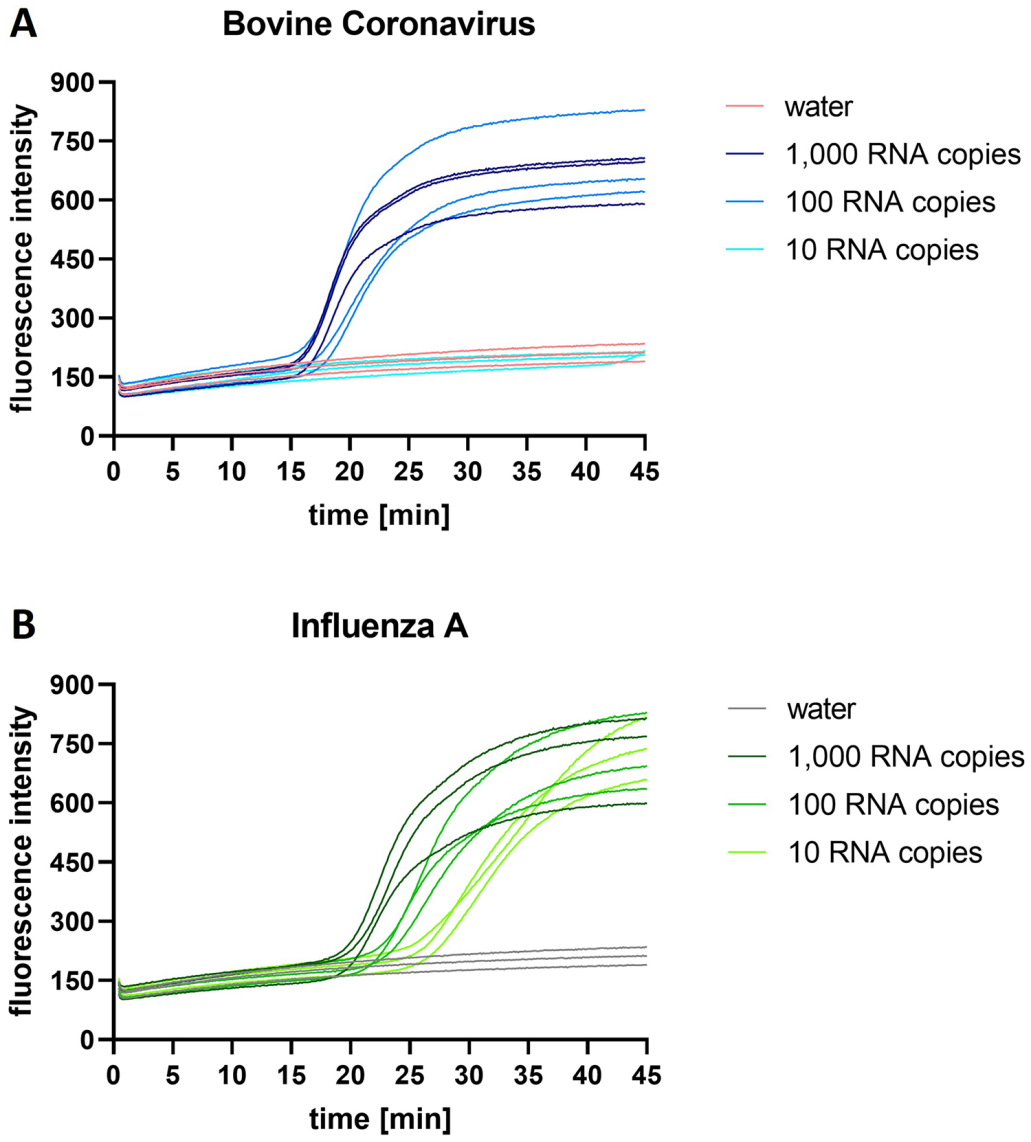
Quantitative RT-LAMP for bovine coronavirus and influenza A virus. RT-LAMP amplification curves for **A** bovine coronavirus and **B** influenza A RNA dilution series detected with LightCycler

The replicates of 1.000 copies and 100 copies of BovCov RNA were positive in quantitative RT-LAMP after an incubation time between 15–18 min (Fig. 3A). The replicates of 10 copies BovCov RNA remained negative. All concentrations of InfA RNA (1,000 copies, 100 copies, and 10 copies) were detected after an incubation time between 19–27 min (Fig. 3B).

**Fig. 4:**
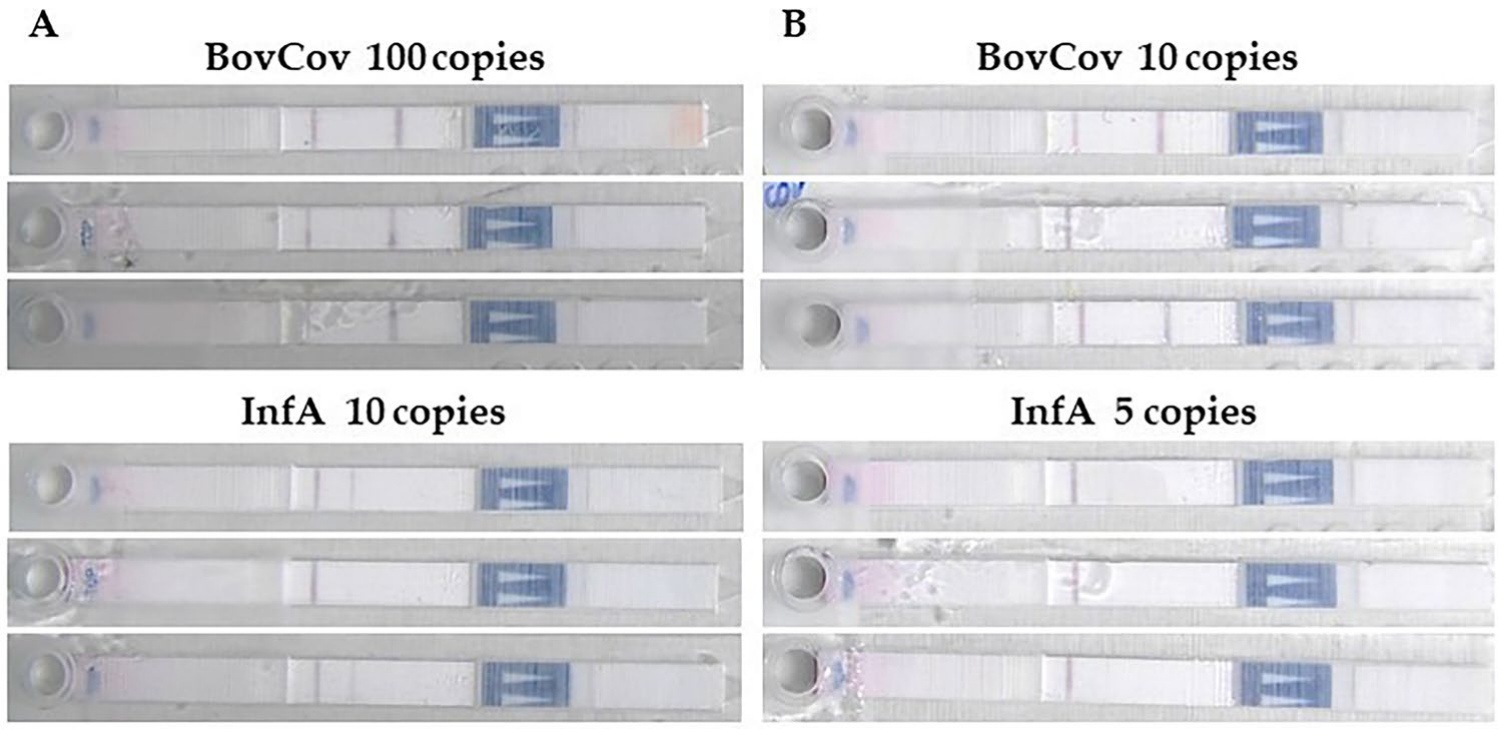
LoD for RT-LAMP device for bovine coronavirus and influenza A virus.** A** Three positive replicates for 100 RNA copies of BovCov and 10 RNA copies of InfA representing the LoD of the respective LAMP reaction; **B** Two positive replicates (top, bottom) and one negative replicate (middle) for BovCov LAMP, two weak positive replicates (top, bottom) and one negative replicate (middle) for InfA LAMP

The same samples were tested with the duplex RT-LAMP device. For BovCov RT-LAMP, a LoD of 100 RNA copies (equivalent to 2,000 copies/ml) was determined with three positive replicates showing intense test bands (Fig. 4A), confirming the results of the quantitative RT-LAMP. For 10 RNA copies, still two out of three replicates were positive (Fig. 4B).

For InfA RT-LAMP (consistent with quantitative RT-LAMP), a LoD of 10 RNA copies (equivalent to 200 copies/ml) was determined with three positive replicates, two showing a moderate test band and one showing a weak test band (Fig. 4A). For 5 RNA copies, two out of three replicates were positive with a weak test band (Fig. 4B). The intensity of the InfA bands should be improved in further development for better read-out, either by adjusting the LAMP reaction conditions or LAMP product binding during LFT. However, the difference between a positive and a negative result was easier to observe by eye than indirectly through photographic images of the result.

## Discussion

The most widely used PoC tests for respiratory viruses are rapid antigen tests that detect viral proteins. These tests are affordable, simple to use and give quick results, but can have low sensitivity and hence present lower negative predictive values than nucleic-acid amplification assays (Dinnes et al. 2022). On the other hand, nucleic acid amplification-based diagnostic tests (e.g., PCR or LAMP) have high sensitivity and high specificity. However, such molecular assay-based tests that detect viruses are slower than antigen tests and are more equipment intense. To address the shortcomings of these different test categories, we created an integrated duplex RT-LAMP device that can be performed without additional equipment. That is, our device was developed to demonstrate the feasibility of a test that better balances the trade-off of device characteristics for a diagnostic application to respiratory viruses.

We showed that integrated duplex RT-LAMP can detect and distinguish between two different respiratory viruses on a single microfluidic chip with LoDs of 2,000 copies/ml for BovCov and 200 copies/ml for InfA. This is in the same range of LoDs of quantitative RT-PCR assays for SARS-CoV-2 like OmniSARS2 (Carvalho-Correia et al. 2021), Erba Molecular ErbaMDx SARS-CoV-2 RT-PCR Kit (Jayakody et al. 2022), or 13 other kits compared by Yang et al., ranging from 68 to 2,264 copies/ml (Yang et al. 2021). Also, compared to other integrated RT-LAMP PoC tests for respiratory viruses (SARS-CoV-2 and Influenza A and B Viruses) our set-up showed significantly better LoD, e.g. Lee et al. showed LoD of 50,000 copies/mL. Moreover, our duplex LAMP system works without an expensive real-time PCR device or other complex devices. In addition, combining the time for LAMP reaction (30 min) and LFT (20–30 min), the total assay time of the duplex RT-LAMP device is 50–60 min – less than half of the time a standard PCR takes (2–3 h). Thus, the duplex RT-LAMP device presented here is as sensitive as gold-standard RT-PCR, but twice as fast, and does not require further special laboratory devices.

Still, some features of our device prototype would need further development. The microfluidic flow of the reagents was disturbed in some chips by minor irregularities of the chips surface in rapid prototypes. It may happen because of the large channel structures that are required by the implemented molecular assay. This provides a high degree of deformation of the material during molding and it is associated with more problematic demoldability. Finally, it results in relatively high manufacturing tolerances in planarity, which explains the defective chips. To counter this, it is possible to switch to a manufacturing process has better tolerances and keep the design. Another option is to slightly modify the chip design and keep the current rapid prototyping manufacturing process.

Comparing the LFT bands, the BovCov test lines were intense, so it was easy to distinguish between positive and negative results. In contrast, the InfA test lines were weaker and thinner, so they need to be improved for better read-out. The amount of LAMP product should be increased by adjusting primer concentrations and reaction parameters. Also, a different primer label for the binding of the InfA LAMP product could be tested. In addition, a clearer surface would improve the general read-out of the LFT, that was hindered by the slightly opaque material - a current limitation of the rapid chip prototyping method from unpolished 3D-printed forms.

Our presented integrated RT-LAMP device can be extremely useful when different respiratory viruses are co-circulating and the testing of patients presenting with acute respiratory illness is needed. Also, it is advantageous to rule out bacterial respiratory tract diseases, since in many cases it is hard to differentiate it from viral infection based on clinical symptoms. It is important to confirm viral disease in order to avoid the unnecessary prescription of antibiotics that could contribute to worsening antimicrobial resistance. In addition, the multiplex panel could be extended with more primer sets to test for other respiratory viruses or specific bacterial pathogens.

The overall innovativeness of the system is its fully enclosed and instrument-free format, i.e. not only the reaction but also the detection including the storage and dispensing of the detection buffer are realized on the single chip, which not only simplifies the handling but also prevents cross-over contamination during the tests.

The realization of this format in a microfluidic chip demanded a few special features in the chip design that ensure a smooth, reproducible, bubble-free filling of the system, fluidic stability during the heating step (no efflux or reflux to the undesired channels and chambers, no pre-term wetting of lateral flow test), and a reliable activation of lateral flow test (which is a particular challenge in an enclosed format, as any imprecisions in chip design either impair the capillary flow on the test strip or create a by-pass and thus would affect the detection efficiency). We have developed a chip design that enables these functionalities without instrumental fluidic control, and, moreover, without active valves or other active mechanical elements on the chip. This system concept enables its cost-saving single-piece manufacturing and makes it a promising diagnostic tool especially in the areas where the pricing for diagnostic devices is a particularly limiting factor.

We have also looked into other published multiplex isothermal amplification methods integrated on microfluidic platforms. Most of them present similar limits of detection, and some of the studies showed tests with capabilities for detecting two or more targets (Chen et al. 2021, 2022; Chen et al. 2021; Wang et al. 2022). However, these microfluidic systems usually needed additional equipment, an electricity source or had longer turnover times, which is not the case in our developed solution. The main drawback described in the literature were that in-house made microfluidic chip-based detection has several challenges when applied on-site and in clinical settings. Firstly, the translation of the R&D systems into clinical practice e.g., via clinical studies, is expensive, because it requires complex chip fabrication, and time-consuming integration of primers and reagents. Secondly, comparability and sharing of data is a problem among different laboratories due to equipment and processing method differences. Thirdly, there are no unified standards for sample preparation and detection, leading to reproducibility issues. Finally, integrating and automating multiple processing links in pathogen detection on microfluidic chips has not yet been achieved (Gao et al. 2022). With our device, we present a fully integrated test format with minimum handling and an instrument-free detection, that reduces any biases in sample processing and interpretation of the results. Moreover, the in-house fabrication of our device is relatively simple and enables scaling e.g., to the needs of R&D studies. Thus, we believe that our integrated system for the laboratory-independent multiplex detection of pathogens can become a feasible tool in PoC diagnostics of infections.

## Materials and methods

### LAMP primer design for bovine coronavirus and influenza A virus

LAMP primer sets for both targets were designed using PrimerExplorer V5 (Eiken Chemical Co. LTD, Tokyo, Japan). N-gene of bovine coronavirus (European Virus Archive Ref-number: 022 V-04370) and M-gene of influenza A/X-31 virus (GenBank number: DQ874879.1) were chosen as target sequences. The sequences of the LAMP primer sets are listed in Table 1.

**Table 1:** LAMP primer sequences for bovine coronavirus and influenza A/X-31 virus

**target**	**LAMP primer**	**5’ label**	**Sequence**
bovine coronavirus (N-gene)	F3	-	CAAGGTGTGCCTATTGCA
	B3	-	TCAGCCTGGTTACTAGCG
	FIP	-	TCGGCTGTTTTAAAAGAACGTCTGGTCCCAGCTACTGAAGCT
	BIP	-	GCTGCCACGATGGTATTTTTACTACTCCGTCAGTATCGGTG
	LF	biotin	GTGTCTGTACCAGTACCCCTT
	LB	FAM	TCTCGGAACAGGACCGCATG
Influenza A/X-31 virus (M-gene)	F3	-	TCCCAGCATCGGTCTCAT
	B3	-	CCAGCACTGGAGCTAGGA
	FIP	-	GCCTTAGCTGTAGTGCTGGCTGGCAAATGGTGACAACAACC
	BIP	-	GGATCGAGTGAGCAAGCAGCACCCAATGGTTCTCATCGCT
	LF	FAM	CTGTTCTCATGTCTGATTAGTGGAT
	LB	digoxigenin	CCATGGAGGTTGCTAGTCAGGC

For lateral flow assay, LF primer of the bovine coronavirus (BovCov) primer set was labeled at the 5′ end with biotin and LB primer with 6-carboxyfluorescein (FAM). LF primer of the influenza A/X-31 (InfA) primer set was labeled with FAM and LB primer with digoxigenin. Primers were synthesized by Eurofins Genomics Germany GmbH (Ebersberg, Germany).

### Isolation and purification of viral RNA from cell culture supernatant

Bovine coronavirus was obtained from European Virus Archive (Marseille, France). Influenza A/X-31 virus was obtained from the Robert Koch-Institute (Berlin, Germany). Virus RNA was isolated from frozen cell culture supernatant using Monarch Total RNA Miniprep Kit (New England Biolabs GmbH, Frankfurt am Main, Germany). Copy numbers of isolated RNA were determined using two-step reverse transcription droplet digital PCR (data not shown).

### Lyophilization of RT-LAMP reagents and chip assembly

RT-LAMP reagents for a 50 µl reaction volume were freeze-dried in the reaction chamber of the chip. The RT-LAMP mix contained 12.5 µl of lyophilization-friendly WarmStart LAMP 4X Master Mix that includes reverse transcriptase (New England Biolabs NEB, Frankfurt am Main, Germany), 5 µl of each of the 10X primer mixes for BovCov and InfA (10X concentration of the primers in each mix: F3 & B3 = 2 µM; FIP & BIP = 16 µM; LF & LB = 4 µM), 12.5 µg BSA (Sigma Aldrich, Germany), 6% (v/v) trehalose (Sigma Aldrich, Germany), and 0.1 mM phenol red (Carl Roth, Germany). Phenol red was added for the visualization and monitoring of fluidic processes on the chip and played no functional role in LAMP reaction.

The lyophilization was performed in a freeze-dryer Christ Epsilon 2–6 (Christ, Germany). After lyophilization, a LFT with two detection lines, one binding to biotin and the other to digoxigenin (Amodia, Braunschweig, Germany) was placed in the chip, and the bottom surface of the chip was sealed with ThermalSeal RTS sealing foil (Excel Scientific, Victorville, USA). A blister filled with 350 µl double-distilled water (microfluidic ChipShop, Jena, Germany) was mounted over the dedicated area using an O-shaped piece of a double-sided sticky tape. The chips were stored at 4 °C until use.

### LAMP-on-chip assay

Lyophilized reagents in the chips were resolved by adding 65 µl of a sample. Samples consisted of the respective RNA dilution in PCR-grade water (Jena Bioscience GmbH, Jena, Germany) and 0.1% (v/v) Triton X-100 (VWR, Darmstadt, Germany) to reduce surface tension, thereby facilitating microfluidic flow. Absolute copy numbers were calculated for a volume of 50 µl, representing the total volume of the reaction chamber. Inlet and outlet were sealed with gas permeable adhesive Air-O-Seal (4titude, Berlin, Germany). The LAMP reaction was performed for 30 min on an in-house-built electricity-free heating system producing a consistent temperature between 72 °C and 60 °C (unpublished data), optimal for *Bst 2.0* polymerase as stated by NEB.

Subsequently, LFT-based detection of the LAMP product was initiated by pressing the water-filled blister until the reaction mixture reached the strip. The result of the LFT was ready for read-out after 20–30 min and stable for several days.
